# Supplementary material for: Use of Antibiotics among Residents Living Close to Poultry or Goat Farms: A Nationwide Analysis in The Netherlands
Source: Antibiotics (Basel). 2021 Nov 4;10(11):1346. doi: 10.3390/antibiotics10111346 (PMC8614970; doi:10.3390/antibiotics10111346)
Supplement: Supplementary file 1 [file antibiotics-10-01346-s001.zip › antibiotics-1433675-supplementary.pdf]

Supplementary Materials

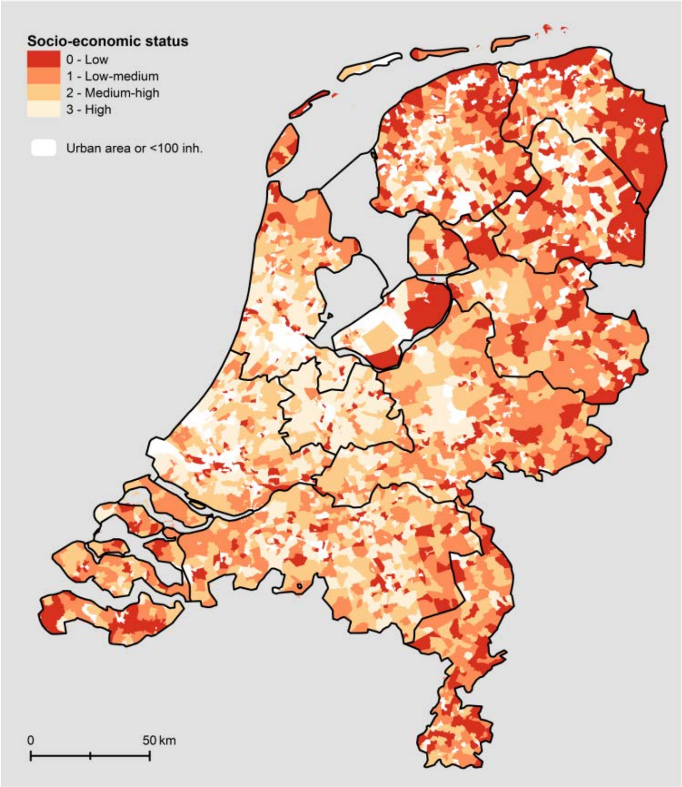

Figure S1. Socio-economic status score at 4-digit postal code level in The Netherlands in 2014 (0: low (score < -0.22), 1: low-medium (-0.22 ≤ score < 0.42), 2: medium-high (0.42 ≤ score <0.96) and 3: high (score ≥ 0.96)). Urban areas and areas with less than 100 inhabitants are displayed in white.

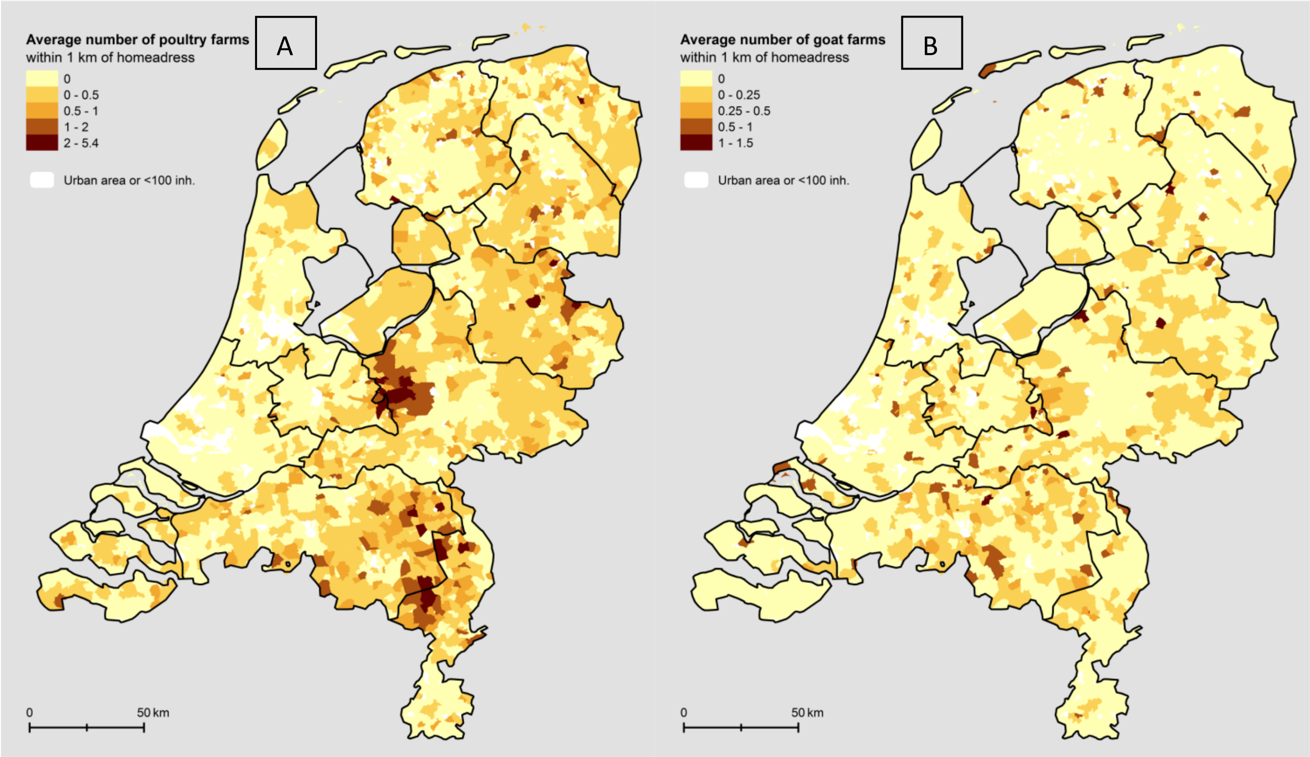

Figure S2. Average number of livestock farms within 1 km of home address in the Netherlands in 2015. **A.** poultry farms. **B.** Goat farms. Urban areas and areas with less than 100 inhabitants are displayed in white.

Table S1. Associations between presence of poultry and goat farms and antibiotic use per type of antibiotic from negative binomial regression, stratified by season

	**Winter months$**		**Other months$**	
**Poultry farm presence**	**Crude ratio* [95%CI]**	**Adjusted ratio** [95%CI]**	**Crude ratio [95%CI]**	**Adjusted ratio [95%CI]**
Amoxicillin	1.09*** [1.05-1.13]	1.06 [1.03-1.09]	1.07 [1.03-1.10]	1.04 [1.01-1.07]
Doxycycline	1.15 [1.10-1.20]	1.10 [1.06-1.15]	1.16 [1.11-1.21]	1.11 [1.06-1.16]
Co-amoxiclav	1.09 [1.05-1.14]	1.05 [1.02-1.09]	1.07 [1.04-1.11]	1.05 [1.02-1.08]
Nitrofurantoin	1.06 [1.02-1.11]	1.00 [0.97-1.05]	1.07 [1.02-1.12]	1.00 [0.96-1.05]
**Goat farm presence**	**Crude ratio [95%CI]**	**Adjusted ratio [95%CI]**	**Crude ratio [95%CI]**	**Adjusted ratio [95%CI]**
Amoxicillin	1.04 [1.00-1.09]	1.01 [0.97-1.05]	1.04 [1.00-1.09]	1.02 [0.99-1.06]
Doxycycline	1.08 [1.02-1.14]	1.04 [0.99-1.09]	1.06 [1.00-1.12]	1.02 [0.97-1.08]
Co-amoxiclav	1.05 [1.00-1.10]	1.02 [0.98-1.06]	1.00 [0.96-1.05]	0.98 [0.95-1.02]
Nitrofurantoin	1.09 [1.03-1.16]	1.04 [0.98-1.10]	1.08 [1.03-1.15]	1.03 [0.98-1.09]

In all models, PC4 was included as random factor. CI: Confidence Interval; PC4: 4-position Postal Code.
* Exponential of the estimate; ** Corrected for SES, age, gender, smoking and presence of goat/poultry farms;
*** Interpretation ratio: the amoxicillin use was 9% higher in the UDPs with poultry farms; $ Winter months: November to March; other months: April to October.

Table S2. Associations between presence of poultry and goat farms and antibiotic use per type of antibiotic from negative binomial regression, for livestock dense area only.

Poultry farm presence	Crude ratio* [95%CI]	Adjusted ratio** [95%CI]
Amoxicillin	1.07*** [1.03-1.11]	1.06 [1.03-1.10]
Doxycycline	1.07 [1.02-1.13]	1.05 [1.00-1.10]
Co-amoxiclav	1.07 [1.03-1.12]	1.05 [1.01-1.09]
Nitrofurantoin	1.09 [1.03-1.15]	1.00 [0.95-1.05]
**Goat farm presence**	**Crude ratio [95%CI]**	**Adjusted ratio [95%CI]**
Amoxicillin	1.04 [1.00-1.08]	1.01 [0.98-1.05]
Doxycycline	1.02 [0.97-1.08]	1.00 [0.95-1.05]
Co-amoxiclav	1.03 [0.98-1.08]	1.00 [0.96-1.04]
Nitrofurantoin	1.09 [1.02-1.16]	0.96 [0.90-1.01]

In all models, PC4 was included as random factor. CI: Confidence Interval; PC4: 4-position Postal Code.
* Exponential of the estimate; ** Corrected for SES, age, gender, smoking and presence of goat/poultry farms;
*** Interpretation ratio: the amoxicillin use was 7% higher in the UDPs with poultry farms.

Table S3. Associations between presence of poultry and goat farms and antibiotic use per type of antibiotic from negative binomial regression, for female gender only.

Poultry farm presence	Crude ratio* [95%CI]	Adjusted ratio** [95%CI]
Amoxicillin	1.06*** [1.03-1.09]	1.04 [1.01-1.07]
Doxycycline	1.12 [1.07-1.17]	1.09 [1.05-1.13]
Co-amoxiclav	1.09 [1.05-1.13]	1.06 [1.03-1.10]
Nitrofurantoin	1.08 [1.04-1.13]	1.03 [1.00-1.07]
**Goat farm presence**	**Crude ratio [95%CI]**	**Adjusted ratio [95%CI]**
Amoxicillin	1.02 [0.98-1.06]	1.02 [0.98-1.06]
Doxycycline	1.02 [0.97-1.07]	1.01 [0.99-1.06]
Co-amoxiclav	0.99 [0.95-1.04]	0.97 [0.93-1.01]
Nitrofurantoin	1.09 [1.03-1.15]	1.04 [1.00-1.08]

In all models, PC4 was included as random factor. CI: Confidence Interval; PC4: 4-position Postal Code.
* Exponential of the estimate; ** Corrected for SES, age, gender, smoking and presence of goat/poultry farms;
*** Interpretation ratio: the amoxicillin use was 6% higher in the UDPs with poultry farms.
